# Augmenting Orbital Debris Identification with Neo4j-Enabled Graph-Based Retrieval-Augmented Generation for Multimodal Large Language Models

**DOI:** 10.3390/s25113352

**Published:** 2025-05-26

**Authors:** Daniel S. Roll, Zeyneb Kurt, Yulei Li, Wai Lok Woo

**Affiliations:** 1 Department of Mathematics, Physics and Electrical Engineering, Faculty of Engineering and Environment, Northumbria University, Newcastle City Campus, College Street, Newcastle-upon-Tyne NE1 8ST, UK; dan.roll@northumbria.ac.uk (D.S.R.); yulei.li@northumbria.ac.uk (Y.L.); 2 Information School, The University of Sheffield, The Wave, 2 Whitham Road, Sheffield S10 2AH, UK; z.kurt@sheffield.ac.uk

**Keywords:** large language models, retrieval-augmented generation, knowledge retrieval, graph databases, orbital debris, space situational awareness

## Abstract

This preliminary study covers the construction and application of a Graph-based Retrieval-Augmented Generation (GraphRAG) system integrating a multimodal LLM, Large Language and Vision Assistant (LLaVA) with graph database software (Neo4j) to enhance LLM output quality through structured knowledge retrieval. This is aimed at the field of orbital debris detection, proposed to support the current intelligent methods for such detection by introducing the beneficial properties of both LLMs and a corpus of external information. By constructing a dynamic knowledge graph from relevant research papers, context-aware retrieval is enabled, improving factual accuracy and minimizing hallucinations. The system extracts, summarizes, and embeds research papers into a Neo4j graph database, with API-powered LLM-generated relationships enriching interconnections. Querying this graph allows for contextual ranking of relevant documents, which are then provided as context to the LLM through prompt engineering during the inference process. A case study applying the technology to a synthetic image of orbital debris is discussed. Qualitative results indicate that the inclusion of GraphRAG and external information result in successful retrieval of information and reduced hallucinations. Further work to refine the system is necessary, as well as establishing benchmark tests to assess performance quantitatively. This approach offers a scalable and interpretable method for enhanced domain-specific knowledge retrieval, improving the qualitative quality of the LLM’s output when tasked with description-based activities.

## 1. Introduction

The accumulation of orbital debris—ranging in size from millimeter-scale paint flecks to entire defunct satellites—has emerged as a critical threat to sustainable space exploration. With over 23,000 cataloged objects currently being tracked in Earth’s orbit and an estimated 100 million additional fragments below the detection threshold of traditional tracking systems, the potential for catastrophic collisions has become a central concern for the aerospace community [1,2]. These objects, traveling at velocities exceeding 7 km/s, can inflict significant damage even when small in scale. The cascading collision scenario known as Kessler Syndrome [3] describes a potential chain reaction where debris collisions generate more fragments, rapidly accelerating the density of debris in key orbital regions and rendering them highly dangerous to operate in. Ensuring the viability of future space missions necessitates robust strategies for debris mitigation, active removal, and crucially, the accurate detection and tracking of existing debris.

Recent advances in artificial intelligence (AI) and computer vision have been harnessed to automate and enhance debris identification, producing a rapidly growing body of literature. Earlier approaches leveraged convolutional neural networks (CNNs) for visual detection from telescope and satellite imagery [4], with models like ResNet and InceptionNet applied to distinguish between debris and active spacecraft [5]. More recently, YOLO-based architectures have shown significant promise for real-time object detection in simulated orbital environments, particularly when applied to range-Doppler data [6]. In radar-focused research, Massimi et al. [7] demonstrated the feasibility of deep learning in radar-based space debris detection by training such YOLO networks on simulated TIRA radar data, outperforming traditional CFAR-based techniques. These methods have also been complemented by Kalman Filters for temporal tracking across frames, forming hybrid pipelines that combine feature detection with predictive tracking [8].

A further promising development has been the exploration of low-resource hardware deployments, such as mounting LiDAR sensors on CubeSats for in-orbit scanning [9]. This approach, combining commercial automotive LiDAR with space-optimized configurations, offers a decentralized and cost-effective means of debris mapping in Low Earth Orbit (LEO), complementing traditional radar-based systems. Despite these advancements, significant challenges remain. Many models are trained on synthetic data or rely on controlled conditions that do not reflect the diversity and unpredictability of real orbital scenes. Moreover, deep learning models are often treated as black boxes, limiting their utility for scientific validation or mission-critical use.

While the benefits of the application of traditional AI methods are well-documented, the potential for natural language-based systems to support this domain remains underexplored. Large Language Models (LLMs), which have transformed fields such as summarization, semantic search, and autonomous reasoning, have seen minimal application in aerospace contexts, and none in multimodal configurations involving both imagery and textual data. This presents an opportunity to investigate how LLMs—especially those capable of vision–language reasoning such as LLaVA [10]—can be adapted to support domain-specific tasks like orbital debris detection, classification, and contextual explanation. Given the technical complexity and data heterogeneity in this field, a hybrid solution that leverages a multimodal LLM’s image-grounded reasoning capabilities, further augmented by domain-specific information retrieved from a structured knowledge graph using Retrieval-Augmented Generation (RAG) [11], may provide a capable method for debris identification. Such a system could integrate summarized academic knowledge with real-time visual analysis to inject up-to-date, relevant context into the model at inference.

The project aims to combine the benefits of multimodal LLMs with retrieved expert knowledge relating to orbital debris detection—an area currently unaddressed in existing literature. At present, the application of language models to the orbital debris domain is novel, and contemporary multimodal technologies have not been researched in the field. The utility of knowledge retrieval on this domain is therefore also untested, identifying a gap in the literature to develop and apply these emerging technologies. The current research therefore proposes a novel application of retrieval-enhanced multimodal LLM technology aimed at the problem of orbital debris identification. By incorporating domain-specific knowledge directly into the language model’s context window via retrieval and prompting the model to interpret and explain orbital debris scenes, this research aims to not only demonstrate technical feasibility but also highlight the utility of LLMs in aerospace applications. This study thus contributes a methodology for AI-assisted space situational awareness and presents a case study exploring the integration of language models and RAG for this purpose. The key contributions of this paper are summarized as follows:

- We propose a novel application of Graph-based Retrieval-Augmented Generation (GraphRAG) in the domain of orbital debris identification, demonstrating its potential to augment the performance of multimodal large language models.
- We design and implement a hybrid architecture that integrates a Neo4j knowledge graph with the LLaVA multimodal LLM, supporting contextual augmentation of image-grounded responses.
- We present a complete end-to-end system pipeline, including knowledge graph construction, query embedding, document retrieval, and prompt engineering for multimodal inference.
- We conduct a case study using the SPARK dataset, evaluating the performance of the GraphRAG-augmented model against both its base version and commercial alternatives (e.g., GPT-4o, Gemini).
- We demonstrate that integrating retrieved domain-specific knowledge reduces hallucinations and qualitatively improves the factual grounding of responses to space imagery-related queries.

## 2. Background

### 2.1. Large Language Models

The progression of Natural Language Processing (NLP), catalyzed by concurrent advancements in artificial intelligence research and technology, has led to its application in a number of key fields [12]. Notably, Pajila et al. explain that NLP is responsible for underpinning machine translation services, enhancing search engine functionality, conducting sentiment analysis to gauge public opinion, and powering virtual assistants and chatbots. Such a varied spread of contemporary applications demonstrates the pivotal modern role of the technology in facilitating advanced human–computer interactions. Central to the advancements in the area are Large Language Models (LLMs), a type of deep learning model that extends the capabilities of NLP through comprehensive pre-training on vast amounts of textual data [13]. LLMs, such as OpenAI’s GPT series, utilize transformer architectures characterized by mechanisms such as self-attention to capture the nuances of human language, allowing for the processing and generation of text that is both coherent and contextually relevant. Incidentally, the training of these models is both data and resource intensive and involves the processing of extensive, diverse collections of language examples to provide the model with the required broad understanding of language and world knowledge. Thus, the development of LLMs has been driven by current advancements in computational power as well as the increasing availability of large-scale datasets [14]. Such extensive training allows the model to perform tasks such as summarization and question answering with proficiency. The current applications of LLMs are vast, including analyzing patient data in healthcare scenarios, predicting financial market trends, and generating content in a wide variety of creative sectors [15]. The inherent adaptability of LLMs to perform disparate tasks without specific retraining highlights the transformative impact of the technology on how machines can process human language. However, a prominent issue is the phenomenon of model hallucination, where the generated response appears true or plausible but is factually incorrect or incoherent. This is thought to arise from a model’s tendency to produce outputs based on learned patterns without grounding in factual data, which can lead to myriad problems with its application or validity, especially in high-risk scenarios such as healthcare diagnosis assistance [16]. Addressing this is pivotal in continuing the development and application of LLMs and has traditionally involved the application of fine-tuning, a process where the model is further trained on a smaller, domain-specific ‘expert information’ dataset to improve its knowledge, thus adjusting the model’s parameters and improving its relevance and accuracy [17]. While effective, fine-tuning is resource intensive and may not fully address problems like bias if the model does not have a way to access the expert information in real time when prompted.

### 2.2. Retrieval-Augmented Generation

An alternative approach to improving model knowledge to increase the quality and validity of its responses is Retrieval-Augmented Generation (RAG). This involves the integration of external data, which can be thought of as expert or domain-informed relevant information, in a way that can be accessed by the LLM in real time [18]. RAG combines the real-time retrieval of this information with the generative capabilities of the underlying model to produce expert-informed responses to the passed prompt without modifying the model’s internal parameters. Typical RAG generally operates by first retrieving the external information, using dense vector search techniques, where the corpus of textual information has been pre-emptively converted into high-dimensional embeddings using pre-trained transformer models such as Sentence-BERT (SBERT) [19]. The embeddings are stored using a similarity search index, a technique used to find data points that are judged to be the most relevant to a given query by measuring the distance or similarity between their vector representations in the high-dimensional space. This is commonly implemented using Facebook AI Similarity Search (FAISS), which uses an approximate nearest-neighbor (ANN) search to locate vectors with the smallest Euclidean distance to the query vector, enabling efficient retrieval of semantically relevant information [20]. Once relevant documents or excerpts are identified, they are appended to the original query as context, augmenting the knowledge of the LLM and facilitating the factual grounding of its resulting response. Therefore, this ensures that the model incorporates externally validated and up-to-date information into its response, beyond the limits of the static training data, reducing the likelihood of hallucinations and allowing for domain-specific adaptation without the need for exhaustive fine-tuning [18].

Although the benefits of incorporating RAG into a language model are well documented, this is not without some limitations [11]. One challenge arises from the fact that the process relies on both the quality and organization of the information corpus, depending heavily on the structure and reliability of the external information database to correctly utilize it. If the information is structured poorly or becomes outdated, this will result in difficulties with retrieving information that is relevant to the query either semantically or temporally, in turn leading to outputs with lower amounts of accuracy or validity. This dependency necessitates continuous maintenance of the knowledge base, ensuring the information is up to date and correctly stored, which may be difficult if the relevant information regularly changes such as in an evolving research domain.

Furthermore, standard RAG methods may often present challenges with retrieval relevancy or errors. When considering a large corpus of external information, it can be difficult for the model to distinguish between closely related topics, resulting in the fetching of information that is tangentially related and therefore contextually plausible, but not directly relevant or the most appropriate for enhancing the response to the specific query. Although research into resolving this problem has been conducted [21], many typical RAG systems often lack iterative reasoning capabilities. Whilst successfully retrieving and presenting information to the user, if the model lacks the capability to understand and interpret the relevancy or utility of it, this can also lead to the usage of information that is semantically similar to the query but not pertinent to augmenting the LLM’s response to it. This is instigated by the way a typical RAG system processes a query, in that the retrieval and generation procedures are commonly conducted in a single pass, and the system gathers information based on the query and responds accordingly. However, when processing a complex or layered question that may require gathering information from multiple sources or reasoning through several steps, the model may not effectively refine its understanding or successfully identify the need for further information, leading to superficial or incomplete answers [22]. Without iterative retrieval and generation cycles, such retrieval inaccuracies can undermine the reliability of the system and reduce its validity.

Recent research into this area has proposed methods that can synergize retrieval and generation in an iterative manner, such as Iter-RetGen [23]. This methodology processes all of the external information as a whole before utilizing a between-steps process to identify what may be needed to complete a retrieval task, providing informative context for retrieving more relevant information. This, in turn, helps to generate a better output in the next iteration, and the authors state that this successfully leverages both parametric and non-parametric information and also performs well on many SOTA benchmark tests. Finally, the integration of retrieval mechanisms also leads to increased system complexity and computational overhead, directly relating to the size of the external corpus, leading to latency issues and affecting the responsiveness of the LLM [24]. Ideally, the system should be able to respond accurately but also promptly, identifying the need for balancing the depth and breadth of retrieval with response speed, a critical challenge in the development of RAG frameworks. While such systems offer significant advantages in enhancing the factual grounding of LLM outputs, addressing these limitations is crucial.

### 2.3. GraphRAG

**Figure 1:**
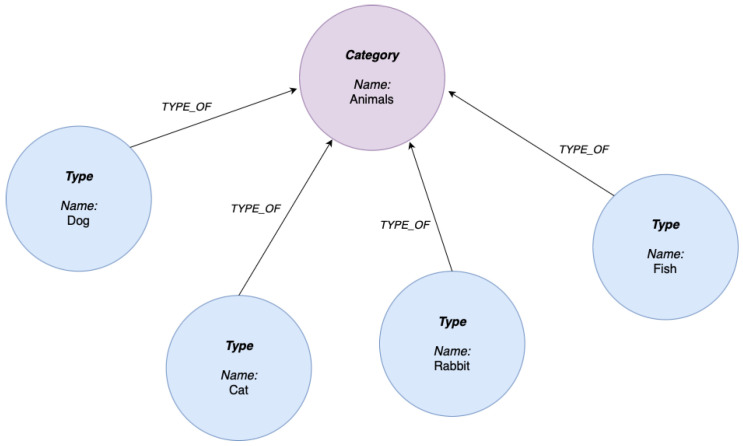
An example of a knowledge graph.

One emerging technology aimed at addressing some of the challenges associated with typical RAG systems is the development of Graph-Based Retrieval-Augmented Generation (GraphRAG). Peng et al. [25] explain that this approach integrates knowledge graphs with an LLM to enhance the retrieval and augmentation processes. A knowledge graph can be considered as a structured representation of information, where nodes in the graph represent entities such as objects or concepts, and the edges between nodes define the relationships between them. Such graphs provide an explicit interpretable structure that allows machines to ascertain and utilize the complex relationships between nodes, making them highly useful for tasks that require contextual depth. Common applications include recommendation systems, semantic search technologies, and question-answering systems. The graph can enable a machine to retrieve contextually relevant, related information as opposed to just individual nodes, providing benefits in these applications. An example of a knowledge graph can be seen in Figure 1.

In GraphRAG, knowledge graphs serve as organized repositories of factual information, while the LLM operates as a reasoning engine to interpret and parse the user’s query, return the relevant knowledge from the graph, and generate a coherent response. The addition of the graph allows the RAG system to connect disparate pieces of information, utilizing the edges to determine connection relevance and therefore attempting to understand the semantic context of the corpus. This can, in turn, increase the accuracy and contextual relevance of the generated response and help to mitigate some of the concerns of hallucination or inaccuracies in the response.

GraphRAG systems tend to follow a typical framework for their design and application [26]. First, the system constructs a knowledge graph from the external corpus. Entities are extracted and assigned as nodes, and the relationships between them are defined. This results in a graph that encapsulates the semantic structure of the overall corpus, providing an efficient and comprehensive summary of the information contained within the text or documents. Then, when a user queries the LLM, the system identifies relevant information in the form of sub-graphs or individual nodes pertaining to the query and retrieves the associated information before passing it back to the prompt. This information, in the form of interconnected entities and relationships, is transformed into a state that can be understood by the LLM using linearization and serialization techniques. This restructured input is then used to generate contextually accurate responses that incorporate the knowledge of the corpus, facilitating outputs grounded in informative context.

As discussed by Peng et al. [25], the integration of knowledge graphs into RAG offers several key advantages over the typical framework; primarily, the enhanced contextual understanding allows the system to better respond to complex queries that may require an understanding of related or interconnected concepts, which allows for more nuanced and accurate responses. This, in turn, leads to improved explainability of such responses, as the explicit, structured nature of knowledge graphs offers a level of traceability. The system can be designed to allow the user to follow the connections and therefore relationships within the graph that are used to augment the response, leading to a degree of transparency that may help to instill trust in the generated answer. Finally, the use of GraphRAG may help to further reduce the occurrence of hallucinations, a key problem in the domain where the LLM responds with information that appears factually correct but is not supported by any actual data. By grounding the responses using the structured knowledge graph, which directly stores domain-specific, correct external knowledge, the frequency of the model producing a hallucination can be reduced. Furthermore, emerging research has demonstrated the efficacy of the GraphRAG approach.

### 2.4. Related Work

Peng et al.’s [25] survey of available GraphRAG literature concluded that leveraging structural information across the entities of the graph not only enables more precise retrieval but can also vastly improve the generation capabilities of the LLM just by capturing relational information. The report formalizes the workflow into a three-stage process beginning with graph-based indexing of the external corpus, which is then used for graph-guided retrieval to catalyze graph-enhanced output generation. The authors do concede that there are challenges relating to the scalability of graph-based retrieval methods due to their computational complexity, and they identify a necessity for heterogenous, well-structured information corpuses. This implies attempting to improve efficiency and aiming to standardize data representation may be important targets for future research in the field.

Adding to this, Hu et al. [27] implemented a graph-based RAG (GRAG) as an attempt to overcome the limitation of naïve RAG systems’ tendency to focus on specific documents and overlook the inter-relevance of networked documents. The authors emphasize the importance of sub-graph structures, explaining that their retrieval over discrete entities maintains an awareness of graph topology. Using a four-stage process, the proposed system first indexes k-hop ego graphs, then it retrieves sub-graphs relevant to the query, prunes these to remove irrelevant entities, and finally uses the pruned textual sub-graphs to generate a response. Hu et al. posit that by integrating both the textual and topographical information, the system produces more contextually accurate and factually coherent responses. Extensive testing on reasoning benchmarks demonstrated that GRAG outperforms current typical RAG methods, effectively mitigating hallucinations in multi-hop reasoning tasks. However, the authors also acknowledged challenges in efficiently retrieving fully optimal subgraph structures. This is explained as occurring as a result of the computational complexity inherent in traversing large graphs, identifying a need for approximation methods to balance applicability and performance.

A notable advancement is the recent Graph Foundation Model for RAG (GFM-RAG) proposed by Luo et al. [28]. This framework adapts the GraphRAG process by including a graph neural network designed to reason over graph structures, aiming to capture complex query–knowledge relationships and mitigate the noise or incompleteness often found in the knowledge graphs. Using a two-stage process, the GFM-RAG was trained on large-scale datasets, comprising sixty knowledge graphs with over 700,000 documents. The authors report that this led to impressive performance as well as generalizability of the model, stating that it is the first graph foundation model that is applicable to unseen datasets without the need for fine-tuning. During testing, the model was applied to both multi-hop question-answering (QA) datasets as well as RAG-specific datasets and achieved state-of-the-art performance. The authors note that the model also maintained efficiency and alignment with neural scaling laws, indicating the potential of the framework for further refinement and application.

Collectively, the available literature highlights the potential for GraphRAG systems to enhance LLM performance through the integration of more structured external knowledge representations. However, they identify key challenges such as increased computational cost and complexity, scalability issues, aand a need for ongoing maintenance of the associated knowledge graphs. Furthermore, the above literature focuses on the implementation of GraphRAG for textual purposes such as QA, with contemporary research suggesting its benefits with regards to facets such as complex reasoning and knowledge planning [29]. With the emerging field of multimodal LLM development becoming more relevant in many sectors, implementing GraphRAG that can assist in both textual as well as visual applications is an important consideration. For example, a common task for multimodal LLMs is to receive an image as input and explain the content or context of said image. Attempting to include an external corpus containing domain-specific information relevant to the images may augment the generated descriptions of the image in the same way it improves the quality of solely textual query outputs. As such, the current research aimed to implement a GraphRAG system using Neo4j, a high-performance native graph database designed for storing and querying interconnected data [30], and incorporate this into a multimodal LLM—the Large Language and Vision Assistant (LLaVA) [10]—for the purpose of generating augmented image explanations. This system can then be queried with orbital debris images, tasked with identifying, explaining, and offering background information on the image as a trial to ascertain if the inclusion of GraphRAG can assist in basic multimodal tasks.

## 3. Technical Overview

This project utilized the Large Language and Vision Assistant (LLaVA) as its core LLM model. LLaVA is a multimodal language model developed by Liu et al. [10] that is based on a combination of the Large Language Model Meta AI (LLaMA) open-source LLM [31] and visual encoders derived from technology such as Contrastive Language–Image Pre-training (CLIP) [32]. LLaVA represents an advanced multimodal LLM that is capable of processing both text and image inputs simultaneously.

### 3.1. LLM Architecture

Large language models, such as the underlying architecture used in LLaVA, operate on the premise of distributed representations of language where words, phrases, and sentences are encoded as high-dimensional vectors in a continuous vector space. Representations are learned from large-scale textual corpora during training, where unsupervised learning objectives are utilized to encourage any semantic and syntactic relationships between words to be reflected in geometric proximity within the embedding space. LLMs are built upon the transformer architecture outlined by Vaswani et al. [33], which utilizes self-attention mechanisms to encode token relationships. This allows each token in a sentence to attend every other token, regardless of position or proximity, contrasting with earlier RNN-based models that struggled with long-range dependencies due to vanishing gradients and other problems. At the core of the transformer is scaled dot-product attention, which enables the model to assess the relevance of words compared to one another. Intuitively, the attention mechanism allows each token in a sequence to ‘attend to’ or focus on other tokens when forming contextualized representations. For instance, in the sequence “the satellite collided with other debris because it was moving erratically ”, the word “it” could refer to “satellite” or “debris”. The model learns to assign higher attention weights to the more relevant word based on the context. This process is underpinned by scaled dot-product attention, defined mathematically as follows:

(1) $$\mathrm{Attention}\left(Q,K,V\right)=\mathrm{softmax}\left(\frac{QK^{⊤}}{\sqrt{d_{k}}}\right)V$$

where $Q\in R^{n\times d_{k}}$ is the matrix of queries, $K\in R^{n\times d_{k}}$ is the matrix of keys, $V\in R^{n\times d_{v}}$ is the matrix of values, and $d_{k}$ is the dimensionality of the key vectors.

However, applying a single attention function may limit the model’s ability to capture different types of semantic relationships. To address this, Vaswani et al. explain that the transformer architecture employs multi-head attention, where the attention mechanism is replicated multiple times in parallel, each with its own learned projections. In multi-head attention, the input embeddings are linearly projected into multiple subspaces, creating independent sets of queries, keys, and values for each attention head. Each head attends to the input using its own scaled dot-product attention computation, potentially capturing a different aspect of the input semantics, such as syntactic, positional, or semantic relations. The outputs of all attention heads are then concatenated and passed through a final linear projection, allowing the model to aggregate diverse contextual information. Mathematically, the multi-head attention mechanism is defined as follows:

(2) $$\mathrm{MultiHead}\left(Q,K,V\right)=\mathrm{Concat}\left({\mathrm{head}}_{1},\ldots ,{\mathrm{head}}_{h}\right)W^{O}$$

where ${\mathrm{head}}_{i}=\mathrm{Attention}\left(QW_{i}^{Q},KW_{i}^{K},VW_{i}^{V}\right)$ is the *i*-th attention head, $W_{i}^{Q},W_{i}^{K},W_{i}^{V}$ are the projection matrices for *Q*, *K*, and *V*, $W^{O}$ is the final output projection matrix, and *h* is the number of attention heads.

A single Transformer block combines multi-head attention with a feed-forward neural network (FNN), interleaved with residual connections and layer normalization. The output of the multi-head attention layer is passed through a position-wise FFN, which applies two linear transformations with a non-linearity in between:

(3) $$\mathrm{FFN}\left(x\right)=\max\left(0,xW_{1}+b_{1}\right)W_{2}+b_{2}$$

where *x* is the input vector to the FFN, $W_{1},W_{2}$ are learned weight matrices, and $b_{1},b_{2}$ are learned bias vectors.

The process is then wrapped in residual connections and layer normalizations to stabilize training and improve gradient flow. The final output of a Transformer block is given as follows:

(4) TransformerBlock(x)=LayerNorm(x+MultiHead(x))+LayerNorm(x+FFN(x))

where *x* is the input to the Transformer block, MultiHead(x) is the result of multi-head self-attention, FFN(x) is the feed-forward output, and LayerNorm(…) denotes layer normalization.

**Figure 2:**
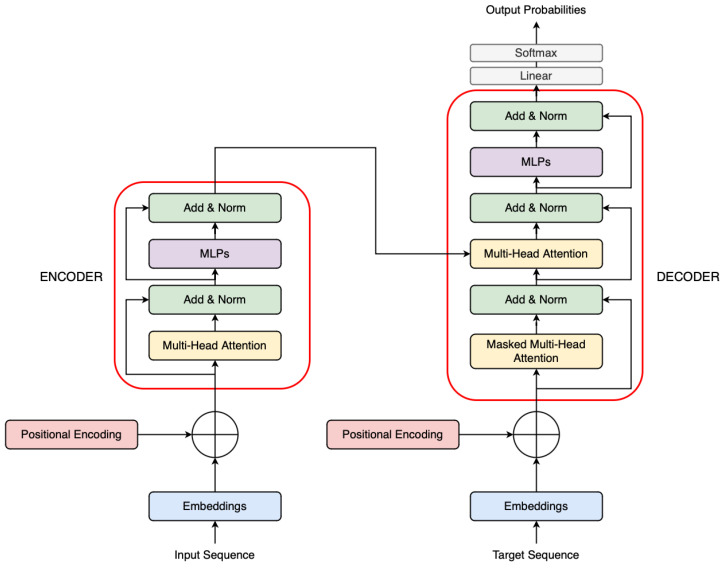
An example of a typical LLM architecture.

These blocks are typically stacked (e.g., 32 layers in LLaMA-2 7B) to form the complete Transformer model, where each layer learns increasingly abstract representations of the input sequence. A visual representation of a typical LLM architecture is shown in Figure 2.

Transformers have demonstrated exceptional scalability and generalization, which is why they are the architecture of choice for models such as LLaMA [31]. The LLaMA architecture can be defined as a decoder-only technology; unlike encoder–decoder models, which separately encode input sequences and decode outputs, decoder-only models process inputs and generate outputs using a single transformer stack. This design is optimized for autoregressive language modeling, where the model learns to predict the next token in a sequence conditioned on previous tokens. The autoregressive language modeling objective is defined as follows:

(5) $$P\left(x\right)=\prod_{t=1}^{T}P\left(x_{t}|x_{<t};\theta \right)$$

where $x_{t}$ is the token at position *t*, $x_{<t}$ are all previous tokens before *t*, and θ are the model parameters.

**Figure 3:**
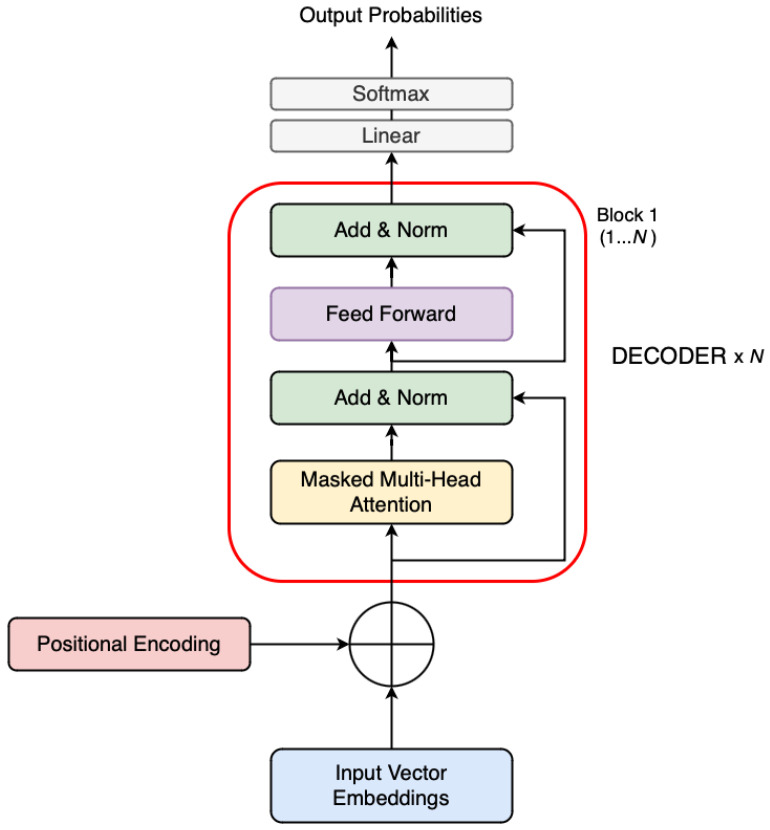
Example of a typical decoder-only LLM architecture.

For each sequence of input tokens, it is passed through an embedding layer and positionally encoded using rotary position encodings (RoPE), which allow for better generalization to longer sequences and improved extrapolation [34]. RoPE modifies the attention mechanism by encoding position information directly into the queries and keys via rotation in complex space rather than using fixed or learnable positional embeddings. The model then processes the sequence through multiple stacked transformer blocks, each consisting of multi-head self-attention and feed-forward sublayers as well as residual connections and layer normalizations. This formulation means the model learns the conditional probability of each token given the sequence history, enabling coherent and contextually aware generation. To prevent information from future tokens from leaking into the prediction, the model applies a causal mask within the self-attention mechanism itself. A visual representation of a typical decoder-only architecture is shown in Figure 3.

### 3.2. Multimodality

While LLMs such as LLaMA provide powerful language modeling backbones, they are inherently unimodal and therefore capable of processing solely text-based input. However, complex real-world applications may require multimodal understanding, particularly the integration of both textual and visual information. LLaVA is a multimodal extension of LLaMA that incorporates visual understanding capabilities via integration with the CLIP vision encoder [10]. When an image is passed as input, it is divided into smaller fixed-size patches and then passed through the CLIP vision encoder. The encoder transforms each patch into a high-dimensional embedding, capturing any rich visual features such as shapes or textures:

(6) $$v=\left[v_{1},v_{2},\ldots ,v_{N}\right],\;v_{i}\in R^{d_{v}}$$

where $v_{i}$ is the *i*-th image patch embedding, *N* is the number of visual patches, and $d_{v}$ is the embedding dimensionality of the CLIP vision encoder.

Each patch embedding $v_{i}$ is then projected into the LLaMA token embedding space via a learned linear projection layer known as the mmproj component:

(7) $${\tilde{v}}_{i}=v_{i}W_{\mathrm{mmproj}}+b_{\mathrm{mmproj}},\;W_{\mathrm{mmproj}}\in R^{d_{v}\times d_{t}},\;b_{\mathrm{mmproj}}\in R^{d_{t}}$$

where ${\tilde{v}}_{i}$ is the projected visual embedding, $W_{\mathrm{mmproj}}$ is the learned linear projection matrix, $b_{\mathrm{mmproj}}$ is the bias term, and $d_{t}$ is the dimensionality of LLaMA token embeddings.

After projection, these visual embeddings are concatenated with the token embeddings $e_{j}$ from the user prompt, forming a single input sequence *x* that combines both modalities:

(8) $$x=[{\tilde{v}}_{1},{\tilde{v}}_{2},\ldots ,{\tilde{v}}_{N},e_{1},\ldots ,e_{T}]$$

where $e_{j}$ is the embedding of the *j*-th input token, and *T* is the number of tokens in the user prompt.

**Figure 4:**
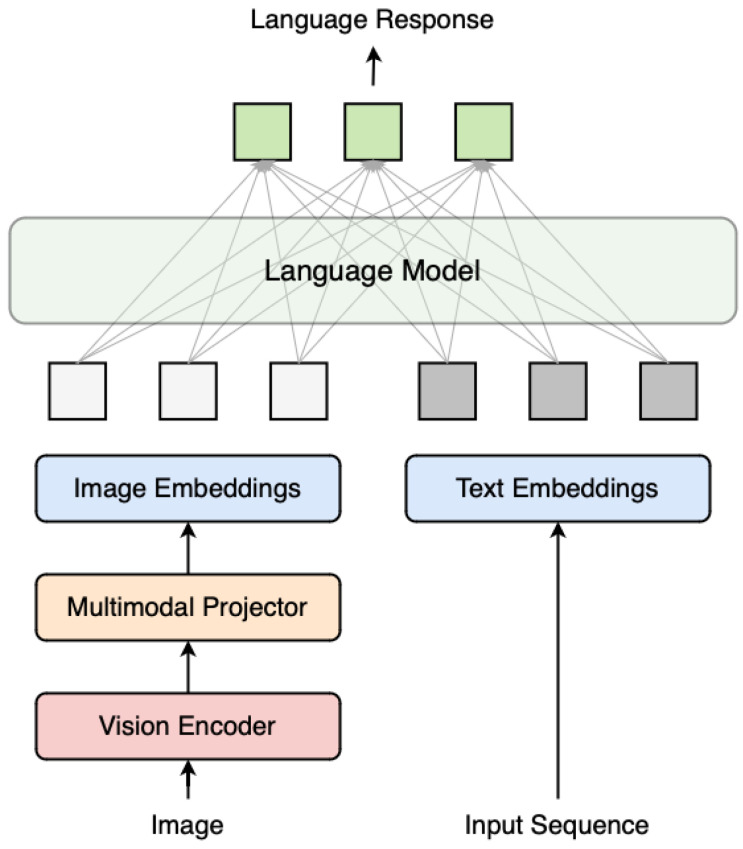
The LLaVA architecture.

This multimodal input is then passed into the underlying transformer decoder. The injected visual embeddings condition the language model to ground its output in visual information, enabling accurate and contextually aware responses to image-based queries. A visual representation of the LLaVA architecture is shown in Figure 4.

### 3.3. Retrieval Augmented Generation

RAG combines parametric knowledge (stored in the weights of an LLM) with non-parametric memory (external knowledge sources such as document databases) [35]. The idea is to retrieve relevant external information based on the user’s query and condition the generation process on this information to improve factual grounding and reduce hallucinations. A user query *q* is first embedded using a sentence or document encoder:

(9) $$\tilde{q}=f_{\mathrm{enc}}\left(q\right)$$

where *q* is the original input query in natural language, $f_{\mathrm{enc}}\left(.\right)$ is the sentence encoder (e.g., MiniLM), and $\tilde{q}$ is the query embedding.

The encoded query is then compared to a set of candidate document embeddings $d_{1},d_{2},\ldots ,d_{n}$ using cosine similarity:

(10) $$\mathrm{sim}\left(\tilde{q},d_{i}\right)=\frac{\tilde{q}\cdot d_{i}}{∥\tilde{q}∥\;∥d_{i}∥}$$

where $d_{i}$ is the embedding of document *i*, ∥.∥ denotes the Euclidean norm, and $\tilde{q}$ is the query embedding.

The top-*k* documents are selected and concatenated with the original query, and this unified prompt *x* is passed to the language model in order to obtain the output *y*:

(11) $$x=\mathrm{Concat}(q,d_{1},d_{2},\ldots ,d_{k})$$

(12) y=LLM(x)

**Figure 5:**
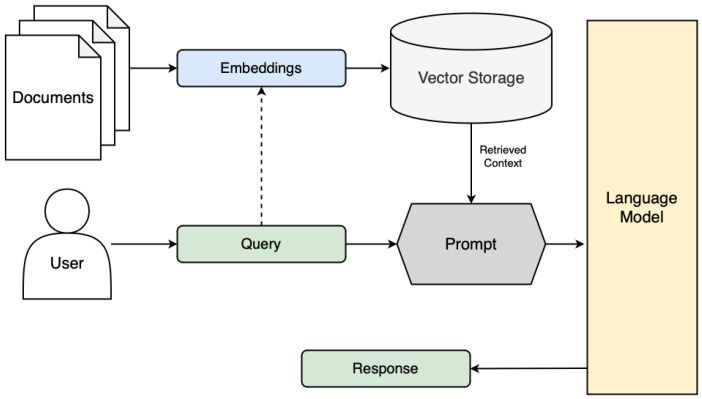
Example of a typical RAG architecture.

Thus, the output has been generated with the additional context available at the point of inference, augmenting the response to the initial query. A visual representation of a typical RAG architecture can be seen in Figure 5.

### 3.4. GraphRAG

Graph-RAG extends the RAG paradigm by replacing the flat corpus with a structured knowledge graph, where information is stored as entities and their relationships [25]. The query embedding is computed in the same way:

(13) $$\tilde{q}=f_{\mathrm{enc}}\left(q\right)$$

where $\tilde{q}$ is the query embedding, $f_{\mathrm{enc}}$ is the embedding model (e.g., MiniLM or BERT), and *q* is the input query in natural language.

This embedding is compared with graph node embeddings $n_{i}$ once again using cosine similarity:

(14) $$\mathrm{sim}\left(\tilde{q},n_{i}\right)=\frac{\tilde{q}\cdot n_{i}}{∥\tilde{q}∥\;∥n_{i}∥}$$

where $\tilde{q}$ is the query embedding and $n_{i}$ is the embedding of node *i*.

Based on this similarity scoring, a subgraph is constructed by selecting the top-*k* nodes and their neighbors, which is serialized into a linear string representation. This is then concatenated with the original query *q* to form an augmented input prompt *x*:

(15) $$x=\mathrm{Concat}(q,f_{\mathrm{linearise}}\left(G_{\mathrm{sub}}\right))$$

where $G_{\mathrm{sub}}$ is the retrieved subgraph relevant to the query, $f_{\mathrm{linearise}}\left(.\right)$ transforms the subgraph into a serialized string, and *q* is the original user query.

**Figure 6:**
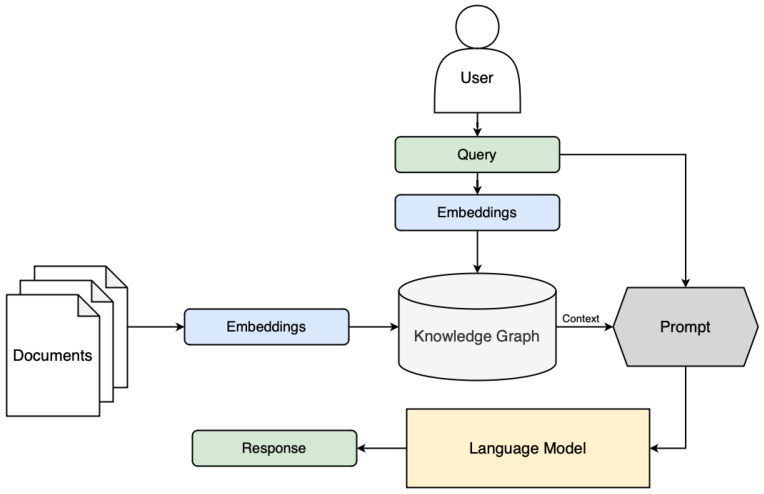
An example of a typical GraphRAG architecture.

This is then passed to the LLM to obtain the output *y*:

(16) y=LLM(x)

where *x* is the augmented input including graph context and the query, LLM(.) is the language model that generates the output, and *y* is the generated response conditioned on both query and graph knowledge. An example of a typical GraphRAG architecture is shown in Figure 6.

## 4. GraphRAG for Orbital Debris

The current widescale application of LLMs to many domains identifies a possibility for their utility in the field of orbital debris. Currently, research applying LLMs to space-centric areas of study such as orbital debris is extremely limited, and the application of multimodal LLMs is nonexistent. Undoubtedly a complex domain, expert knowledge relating to the background and detection methods for such debris is critical in understanding the problem. As such, the current research aimed to integrate GraphRAG within the LLaVA multimodal LLM and aim this technology at the field of orbital debris in a bespoke case study.

### 4.1. Proposed Architecture

The proposed system is designed to enable multimodal LLM inference over a domain-specific knowledge graph, grounded in both image and text inputs. The core idea is to enhance a vision–language model (LLaVA 1.5) with external academic knowledge through the use of a graph-based retrieval-augmented generation (GraphRAG) pipeline. The architecture is constructed in modular stages: it begins by processing a corpus of academic research papers in PDF format, which are automatically parsed using PyPDF2. Each paper is summarized using GPT-4 to ensure coherent and contextually relevant abstracts. Titles are extracted using an additional GPT prompt, and keywords are automatically generated via KeyBERT. These metadata fields (title, summary, and keywords) are then stored in a JSON file, which is used to populate a Neo4j knowledge graph. To enhance the graph’s semantic utility, relationships between papers are also automatically generated using GPT-4, based on comparative reasoning over summaries and keywords. These relationships are stored as directed edges in the graph with type and explanation attributes, forming a rich interconnected corpus of research knowledge.

To support similarity-based retrieval, each summary is embedded using the all-MiniLM-L6-v2 model from SentenceTransformers and stored as a vector attribute on the corresponding node. At runtime, the system accepts a natural language query and an optional image. The model uses the MiniLM to embed the user query and, using cosine similarity, compares this with the node embeddings to identify relevant documents from the graph. The top-k documents, along with their relational context, are appended to the LLM input prompt to provide factual background to the LLaVA model. If the original question fails to retrieve sufficiently relevant documents from the graph, the image can also be described in natural language using GPT-4. This description is embedded with MiniLM and used as a fallback query. This two-stage fallback logic ensures that vague or ambiguous questions can still benefit from visual grounding. Importantly, while the LLaVA model internally uses CLIP for image understanding, these internal image embeddings are not externally exposed, so we rely on the GPT-generated descriptions for graph querying in this instance. While this fallback mechanism enables flexible multimodal retrieval, it introduces an additional layer of abstraction between the image and the graph, which may influence retrieval accuracy. The quality of the GPT-generated image caption plays a crucial role in determining subgraph selection during inference. If the caption is overly generic or contextually misaligned, it may fail to activate semantically relevant nodes in the graph, leading to reduced retrieval precision. Conversely, overly specific or speculative captions could bias the subgraph selection toward niche or irrelevant topics. This introduces a dependency on the generative quality of GPT-4’s captioning, which, although typically adequate, is not immune to hallucination or ambiguity. Future work could explore more robust visual–semantic embedding techniques that directly connect image features to graph structures, possibly through fine-tuned CLIP vectors or multimodal contrastive training approaches. Nonetheless, in the current system, the existing fallback mechanism can still provide valuable context in otherwise underspecified queries.

**Figure 7:**
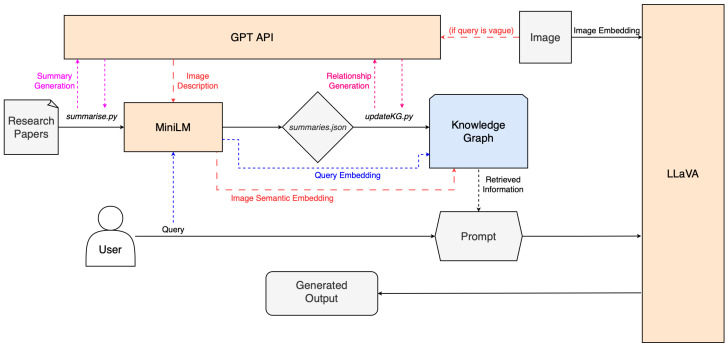
System diagram showing the proposed architecture.

Rather than using the LLaVA command-line binary directly, the system wraps its execution in a Python (v3.11.0) script that programmatically builds the input prompt, runs the model, and captures both the generated response and system runtime statistics (loading, evaluation, and generation times). This design allows the system to maintain compatibility with CLIP-powered LLaVA while still enriching its responses with graph-sourced knowledge. Altogether, the architecture demonstrates a hybrid pipeline combining retrieval-augmented generation, vision–language understanding, and knowledge graph reasoning. The system diagram can be seen in Figure 7.

### 4.2. LLaVA Setup

To enable local and efficient multimodal inference, LLaVA v1.5 was deployed using the lightweight C++ library llama.cpp. This is an open-source implementation of the LLaMA model architecture that is designed for Apple GPU acceleration [36]. As such, llama.cpp is a highly optimized inference framework that facilitates GGUF-quantized LLM execution. This was beneficial to the current work, as it allowed for the local execution of the LLM directly on an Apple Silicon (M2 Max) device running MacOS, without requiring access to cloud-based resources. This aligns with emerging trends in privacy-preserving computation, as explained by Yan et al. [37], which call for models to be deployed on-device. The model, however, was relatively large (7B), and in order to fit this within the hardware limitations of the local machine, quantization was employed. This involves the reduction of precision of the model weights in order to significantly lower the memory footprint and improve execution efficiency [38]. Research into modern quantization methods has proven they are able to preserve the generation capabilities and language understanding of the model while reducing its size significantly [39]. For this implementation, the model was quantized with Q4-K, a 4-bit quantization approach that offers a balance between performance and accuracy. The specific LLaVA model file, ggml-model-q4_k.gguf, was compatible with llama.cpp’s quantization-aware GGUF loader and was paired with the multimodal projector file mmproj-model-f16.gguf to support image input alignment for multimodal reasoning. Furthermore, llama.cpp includes support for Metal Backend integration on Apple devices. Metal is Apple’s low-level graphics and compute API, and this can be enabled when building the model to use Metal shaders to offload parts of the LLM computation graph such as attention or MLP layers onto the GPU, yielding substantial performance gains [40]. Additionally, the current llama.cpp resource includes the llama-llava-cli binary file, which enables LLaVA-specific prompts to be processed and executed through terminal commands. By specifying model weights, the multimodal projector, an optional image, and a text prompt with this CLI, the model could be quickly tested directly through the terminal. Once implemented, the model was initially tested for functionality by passing an arbitrary unimodal command using this CLI file. During execution, debug logs confirmed that the model was successfully offloaded, with many layers allocated to Metal-accelerated memory buffers. This enabled inference at an acceptable speed of a few seconds per prompt and confirmed that the base model was sufficiently performing on the local hardware.

### 4.3. GraphRAG Setup

The GraphRAG system developed in this study is designed to incorporate knowledge graph-based retrieval into a multimodal language model workflow, enabling context-aware responses grounded in domain-specific research. The architecture follows the RAG paradigm but with a graph-structured backend. This enables not only semantic similarity-based document selection but also retrieval of interconnected concepts and entities via explicit graph traversal.

At a high level, GraphRAG consists of three primary stages: (1) construction of a knowledge graph from an external corpus, (2) embedding-based retrieval of relevant nodes from this graph using user queries, and (3) prompt augmentation for an LLM using retrieved graph information. The system is designed to be modular and corpus-agnostic, and its scalability is determined primarily by available compute and storage resources, not by design limitations. The knowledge graph can grow dynamically, and its topology reflects the semantic and conceptual relationships between documents, keywords, or extracted entities. This design enables richer context retrieval than traditional flat-vector RAG systems by supporting multi-hop and relation-aware augmentation strategies [25].

#### Corpus Selection

To evaluate this architecture in a controlled setting, a small document corpus was used as a proof-of-concept. Five academic papers focusing on orbital debris and its detection were selected due to their domain relevance and familiarity to the research team. This limited-scale setup allowed for easier debugging and reduced computational overhead during initial development. The selected documents were as follows:

1. “Detecting, Imaging and Tracking Space Debris” by Mehrholz, Leushacke, Flury, Jehn, Klinkrad and Landgraf [41]
2. “A Deep Learning Approach for Satellite and Debris Detection: YOLO in Action” by Ahamed, Bin Syed, Chatterjee and Bin Habib [42]
3. “CosmosDSR - a methodology for automated detection and tracking of orbital debris using the Unscented Kalman Filter” by Roll, Kurt and Woo [8]
4. “Deep learning-based space debris detection for space situational awareness: A feasibility study applied to the radar processing” by Massimi, Ferrara, Petrucci and Benedetto [7]
5. “Space Debris In-Orbit Detection with Commercial Automotive LiDAR Sensors” by Lopez-Calle [9]

While this study was intentionally limited to a small five-paper corpus for controlled evaluation, the system architecture is designed with scalability in mind, although it is pertinent that scaling to larger corpora (e.g., hundreds of papers) introduces both computational and practical challenges. The most computationally sensitive component, and therefore the primary bottleneck when scaling the corpus, is the similarity-based retrieval stage, which uses MiniLM sentence embeddings and cosine similarity search via a linear scan over node vectors. While fast for the small graph produced by the current study, this approach scales proportionally with the number of documents. For larger corpora, this will undoubtedly significantly increase computational efficiency. Substituting this with another approach such as Facebook AI Similarity Search (FAISS) [20], which is an efficient ANN search library optimized for high-dimensional vector comparison, may optimize retrieval times. Additionally, the prompt construction phase, where retrieved nodes and their relationships are formatted for the LLM input, does not scale with the size of the corpus but only with the number of top-k retrieved documents, which remains fixed in our architecture. Thus, even as the corpus grows, the latency incurred during prompt assembly remains stable and predictable. It is also worth noting that the graph’s semantic connectivity, or the number of relationships between documents, could grow exponentially if all papers are densely connected. In order to empirically assess the impact of this, scaling trends could be benchmarked across corpora of increasing size to quantify the trade-offs between retrieval latency, LLM input limits, and relevance quality. Such considerations are highlighted to guide potential future deployments and adoption to other application areas.

The above papers were downloaded and stored locally in a folder within the llama.cpp environment for usage throughout the project. The next step was to create the knowledge graph itself, and this was achieved using the aforementioned graph database software Neo4j [30]. This utilizes a property graph model, where nodes represent entities such as papers or concepts, and edges represent the relationships or interconnections between them. Angles et al. [43] explain that each can hold metadata in the form of key-value properties, allowing for expressive, human-interpretable data modeling. Neo4j was selected for both its maturity and extensive documentation, as well as its compatibility with Python via the py2neo client. Although it can be implemented directly through the terminal, the software was installed via the Neo4j desktop application for MacOS, as this provides a resolute GUI allowing for visual project management and simplified database administration. A dedicated local database was created within the GUI, and it was ensured that the binary protocol for efficient query execution, Bolt, was enabled and listening on the correct port. The use of this protocol allows for low-latency transactional interaction between the graph and any retrieval engines that are developed.

**Figure 8:**
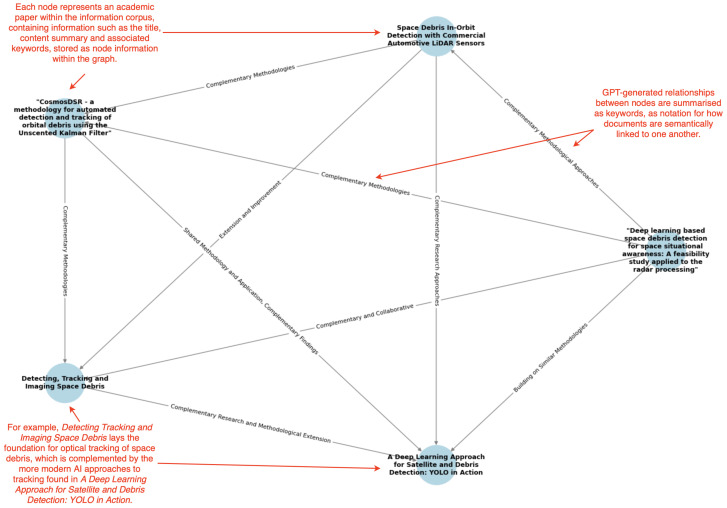
The generated Neo4j knowledge graph.

In order to populate the graph with the downloaded papers, the critical information was extracted and summarized using the aforementioned architecture before being stored in the JSON file. At this stage, a script to visualize the knowledge graph was also created. This again uses the py2neo client to interface with the graph, utilizing a combination of the network and matplotlib libraries to generate a visually appropriate graph displaying all nodes and interconnections. An example of the generated knowledge graph from the above papers can be seen below in Figure 8.

Finally, the script to run the LLM with the added GraphRAG functionality was developed. This was facilitated through the usage of Python’s subprocess library to invoke the llama-llava-cli process within the script itself, allowing for the addition of the relevant RAG components. The arguments are still passed through the terminal, with the script simply replacing the ‘./llama-llava-cli’ portion of the command. The script takes the arguments defined by the user, including the standard model and multimodal projector files, the path to any specified image, the required temperature, and finally their query, and it uses this information to invoke the subprocess. However, the script defines a prompt that will be passed to the LLM, and this therefore allows for interjection within this prompt to augment the response. As outlined in the architecture, the user’s question is first embedded into a vector using the all-MiniLM-L6-v2 model from the sentence-transformers library, a lightweight Bidirectional Encoder Representations from Transformers (BERT) model optimized for sentence-level semantic similarity [44]. The generated vector therefore represents the semantic meaning of the user’s query. The script then pulls all summary fields from any existing Paper nodes in the knowledge graph and encodes these into vectors using the same model. The resulting vectors are stored as properties of the node; thus, by calculating the cosine similarity between the query embedding and each node’s summary embedding, their semantic relevance is quantified. A threshold value was defined, and any papers that exceed this can be considered relevant in terms of context. Therefore, the top-k most similar papers are selected based on this information and provide the baseline for further context construction. For each of these top-ranked nodes, a Cypher query is raised to retrieve all outgoing relationships from that node and collect the summary information of each related node. This information is then appended to the retrieved context before it is all passed to the prompt alongside the user’s original query. This prompt is then passed to the LLM, which generates a response to the query augmented by any information retrieved from the relevant nodes of the graph. Therefore, as the content of the external data is generally outside the model’s pretraining corpus, it should now be able to use this ‘expert insight’ to generate more factual responses grounded in domain-specific research-driven information.

### 4.4. Image Selection

**Figure 9:**
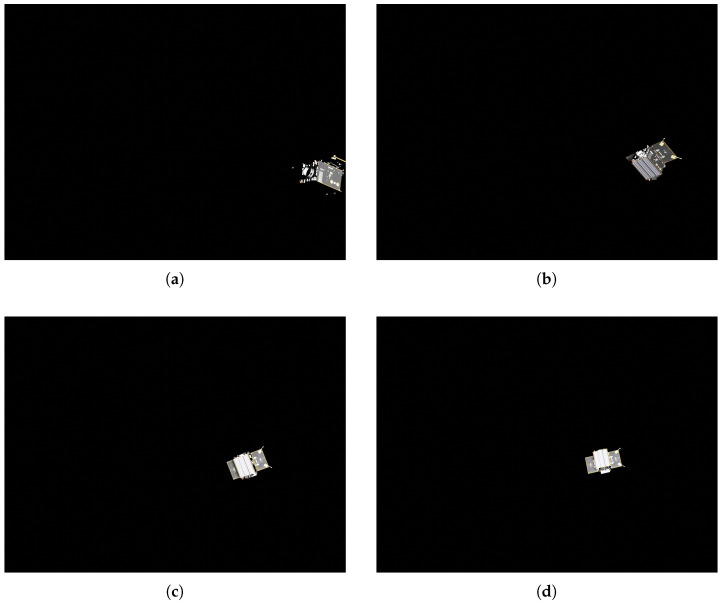
Example images from the SPARK synthetic data-stream ‘GT066’, displaying the Proba-2 Satellite: (**a**) img293_GT066.png; (**b**) img106_GT066.png; (**c**) img077_GT066.png; (**d**) img001_GT066.png.

In order to test the multimodal aspect of the LLM, a test image relevant to the research area was chosen. This was a synthetic image of orbital debris, taken from the established Spacecraft Recognition Leveraging Knowledge of Space Environment (SPARK) dataset provided by Musallam et al. [45]. This is a specialized space object image collection designed for Space Situational Awareness (SSA) tasks. Created under realistic space simulation conditions, it features varied sensing environments and orbital scenarios. It includes images of varied Resident Space Objects (RSOs) against a black background. The decision to use these synthetic data arose from the obvious lack of noise in the images; while not exactly representative of the minimal real RSO images available, it should make the objects more easily distinguishable, enabling the focus to rely on the RAG elements of the framework as opposed to the detection capabilities of the underlying model. Furthermore, while ‘debris’ is often used to define visibly damaged orbital entities, some debris relates to simply defunct satellites or retired spacecrafts that remain in orbit [2]. When considering the problem of residual debris, such objects must also be accounted for. As such, and as no additional context to the functionality of any satellite in the images is given to the model, this research uses the term ‘orbital debris’ to define any object in the images. This was deemed an appropriate method of analyzing how responses may be biased to define debris as solely damaged objects and overlook the possibility that a visually functional yet defunct satellite could also be considered hazardous. Example images from the SPARK dataset, including the test image (b), can be seen below in Figure 9.

However, real-world astronomical imaging, particularly in the context of space debris detection, often encounters several challenges that can impact image quality. One issue involves the inclusion of lens artifacts present within an image, occurring when the optical components of the telescope or camera introduce slight distortions due to reflections from light sources such as the sun, or simply lens misalignment [46]. This often results in fixed-pattern distortions to the image, such as vignetting, overexposure, or sensor glare, which may obscure relevant visual signals in the scene. The field of astronomical image processing is well-developed, however, and research into mitigation techniques is abundant. For example, a common method to remove lens artifacts is median background subtraction, which computes a per-pixel median from a series of consecutive frames and subtracts this background from each image [47]. Another issue is observable noise within images taken in low-light or high-sensitivity conditions, as they can be affected by electronic noise introduced during capture [48]. To reduce this, basic spatial filters such as the Gaussian filter are often applied, smoothing the image via convolution with a Gaussian kernel to suppress high-frequency noise while preserving low-frequency structure [49]. Alternatively, applying Fourier-based filtering to a transformed image provides another approach, isolating and suppressing known noise patterns in the phase or magnitude spectra, particularly where sensor or optical interference dominates [50]. Furthermore, there is also the issue of atmospheric-induced degradation [51], where captured images may suffer from blur, haze, or low contrast due to imaging under poor lighting or inclement weather. Advanced enhancement techniques have been proposed to counteract such degradation. For instance, Tao et al. introduced a visible and infrared image fusion method that enhances space debris surveillance by combining information across spectral bands, improving contrast and perceptual clarity in adverse conditions [52]. Similarly, Liu et al. propose a variational night-time dehazing method, designed for intelligent surveillance tasks, that decomposes low-light hazy images into structural, detail, and noise layers via hybrid regularization. This allows for targeted enhancement of critical features while also reducing noise [53]. When considering the proposed future application of the current research to real images, known issues such as these must be thoroughly assessed in order to carry out adequate preprocessing to mitigate the associated concerns. Although outside the scope of this paper, developing a thorough preprocessing methodology for real astronomical images to be used with the current system is an immediate future aim of this work and must be completed before the current research can be applied to real-world scenarios. Incorporating the above image enhancement techniques into the preprocessing pipeline is essential for improving the visibility and robustness of input data, and this, in turn, should strengthen the performance and reliability of the current system architecture.

### 4.5. Query Selection

For testing the model, alongside the synthetic image, a number of queries were devised to assess the performance with regard to various tasks. These were designed in a mixture of multimodal styles that a typical user of the technology in the field may ask, incorporating elements such as image confirmation, image description, and further knowledge retrieval alongside some hybrid tasks. Although the chosen queries were not selected based on a specific strategy, they were devised following discussions with potential end users (astrophysics researchers and commercial/military orbital analysts) to encapsulate recurring themes in their requirements. Again, due to the preliminary nature of this study, this is a small selection of queries that will need to be significantly expanded in future work. The chosen queries can be seen below in Table 1.

### 4.6. Evaluation Methodology

To assess the quality of responses across all models (LLaVA 1.5, GraphRAG-augmented LLaVA, GPT-4o, and Gemini), a qualitative evaluation based on three key criteria was performed:

- Factual Accuracy: Whether the response contains verifiable, correct information grounded in known scientific literature or domain knowledge.
- Hallucination Presence: Whether the model invents facts, such as nonexistent paper titles, misidentifications, or speculative conclusions unsupported by the input.
- Contextual Relevance: Whether the response directly addresses the query and makes meaningful use of either visual input or retrieved knowledge (in the case of GraphRAG).

All outputs were manually reviewed, and the presence of hallucinations was assessed through comparison with context from the relevant academic databases (Google Scholar, IEEE, arXiv etc.). To evaluate hallucinations more systematically, they were categorized into three established types: factual, identification, and inferential hallucinations. This taxonomy is adapted from prior work on LLM reliability and hallucination research [16,54].

- Factual hallucinations refer to assertions that contradict known truths, such as citing non-existent articles or inventing false information.
- Identification errors arise when models incorrectly label or describe objects, people, or locations, particularly in vision–language settings.
- Inferential hallucinations occur when the model draws speculative conclusions or extrapolates beyond the provided input, despite no visual or textual evidence justifying the leap.

To apply this framework, each model output was reviewed and cross-checked against trusted sources for factual consistency. Identification errors were verified by comparing model statements with actual image content and metadata. Inferential hallucinations were flagged when conclusions were not reasonably supported by the input data (either visual, textual, or retrieved). This allowed for clearer analysis of the types of errors made by each system. In addition, each model’s runtime was noted to provide a basic comparison of computational efficiency. While this evaluation is qualitative, it allows for a clear initial comparative assessment of model behavior in a domain-specific application. Future work will need to include formal benchmarking with domain experts or annotated gold-standard response sets for quantitative scoring, although this is outside the scope of the current paper and is thus an immediate next step for the research.

### 4.7. Results

Each query was passed both to the baseline LLaVA model with no further enhancement and to the GraphRAG-augmented LLaVA, as well as other leading commercial models including Gemini [55] and GPT-4o [56] for comparison. The model output was gathered to compare performance across tasks. Debug logs were used to confirm Metal backend usage for both LLaVA models and offer relevant loading, evaluation, and generation times for further comparison of computational efficiency.

#### 4.7.1. Test 1

The first test comprised a straightforward image confirmation problem. Given that the subject was included in the query, it was hypothesized that both the core model and the GraphRAG-augmented model should perform well on this. The results can be seen in Table 2.

As shown by the above responses, both LLaVA models answered the query with a confirmation of the object. Interestingly, the core LLaVA model responded with a much longer statement, adding potentially unnecessary information. Additionally, the response was less sure with regard to the exact object. Both Gemini and GPT-4o identified the object as a satellite as opposed to debris, inferring functionality from the lack of visible damage, as hypothesized. Gemini misidentified the satellite as Rosetta, when the object displayed in the image was actually Proba-2. The total runtime was shorter for the RAG-augmented model, with disparities potentially caused by the shorter response and the more intense evaluations of the prompt in other models.

#### 4.7.2. Test 2

The second test took this a step further, passing an open-ended query that gave no prior information about the object in the image to assess the image identification capabilities of the model. It was hypothesized that all models should be able to accurately identify a piece of debris/satellite/RSO within the image due to their stance as leading vision language models. The results of this can be seen in Table 3.

The responses here once again offer seemingly valid answers to the given prompt. All models correctly identify the object as an RSO and give some other supporting information. The base LLaVA model speculates on the recent deployment of the satellite, which is not possible to determine from the photo and may represent some fringe information learned from its training corpus. The GraphRAG-assisted model does bring in some language that appears to be directly related to the sentences used in papers retrieved from the knowledge graph. However, this also denotes the object as central in the image, which is not accurate. Although not necessarily a hallucination, this highlights the known challenge of localization within visual models. Gemini once again misidentified the satellite in the image as the Rosetta rather than the correct Proba-2. GPT-4o did ask for further information, but it also mentioned the SPARK project in its response, the dataset the image was taken from. The base LLaVA model had a short runtime, potentially due to the lack of depth within the response, and conversely, a significantly longer runtime was observed from GPT-4o than any other model.

#### 4.7.3. Test 3

The third query passes a hybrid task in which the models are prompted to first confirm the object, then offer and discuss a relevant academic paper on the topic. It was hypothesized that the commercial models would perform this task well due to their extensive pretraining, with the RAG-augmented model hopefully bringing in some detectable relevant information from the corpus. The results can be seen in Table 4.

The responses to this particular query offer some interesting results. While the base LLaVA model successfully confirmed the image as orbital debris and retrieved an academic paper, this specific piece of literature does not exist. Extensive searches of both the paper title and the author’s name in repositories such as Google Scholar returned no related results, identifying that although this response appears a valid and informed statement, it was in fact a hallucination of the model. In comparison, the GraphRAG-augmented model suggested a very well-known publication from the domain [41], which was retrieved directly as a node of the knowledge graph during inference, highlighting the positive impact of incorporating the retrieval technology into the core model. Both Gemini and GPT-4o answered the prompt by finding a relevant paper without hallucination. Both responses were factual and well-constructed, as can be expected of such models. Interestingly, Gemini did erroneously return the first author’s name (Hickson [57]) in Russian. Once again, the runtime of all models was relatively similar, with the commercial models running a few seconds shorter overall.

#### 4.7.4. Test 4

The fourth test probed the models with another hybrid task to describe the risks associated with the object in the image. This represents a multi-hop style question bordering on the necessity of advanced reasoning; in order to successfully answer the query, the model would have to first identify the object, understand the connotations of ‘risk’ in this application, and then retrieve information to explain this. Overall, it was hypothesized that the GraphRAG augmented model should respond better to this question overall by providing specific context from the corpus. The results can be seen in Table 5.

The responses to this prompt were relatively similar for all models, accurately identifying the object as an RSO and touching on some of the associated risks. The LLaVA model’s response had some slight repetition, although the sentiment of this response was practically the same as the GraphRAG-augmented model and sufficiently answered the query. There was some evidence of information from the RAG corpus in the augmented model’s response. Gemini misidentified the satellite as Rosetta again, leading to specific details of this mission erroneously applied to the given image. The GPT-4o model provided a strong response, albeit with the longest runtime.

#### 4.7.5. Test 5

The final query proposed another hybrid task requiring multiple steps to answer successfully, tasking the models with identifying the object and then describing the current state of research literature surrounding it. Based on the results of previous tasks, it was hypothesized that the augmented model’s response would be more accurate and valid for this application than the core model, as well as potentially retrieving specific documents from the corpus when compared to the commercial models. The results can be seen in Table 6.

The responses to this prompt offered some interesting insights, mainly supporting the prior hypothesis. The core LLaVA model attempted to answer the query, though it failed to fully identify the object and gave very broad, nonspecific information that did not really answer the question with any real confidence. In contrast, the GraphRAG model correctly identified the RSO and retrieved insights from the papers within the knowledge graph, generating a response that captured methodologies and technologies used in the corpus’ literature. This did, however, increase the runtime significantly. Gemini once again misidentified the object and, as such, returned information about research on that specific satellite as opposed to the general research area. GPT-4o actually identified the object as a SPARK object; however, it then produced a hallucinated description of what this is instead of presenting the actual SPARK acronym. This, in turn, resulted in a response that was tailored to small satellites in general, which is still within the scope of the question. Quantitatively speaking, the GraphRAG-augmented model produced the most desirable response to this prompt.

### 4.8. Hallucination Analysis

**Figure 10:**
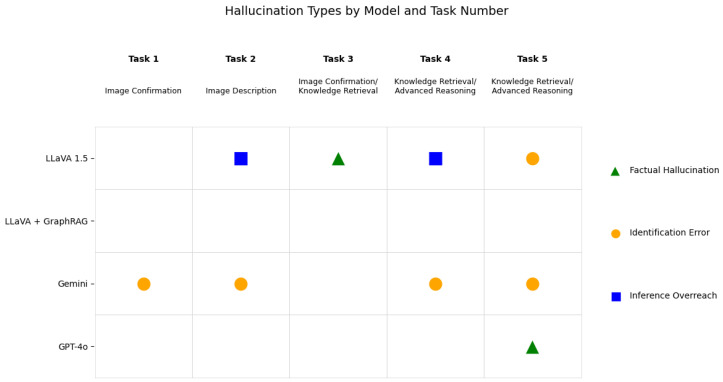
Observed model hallucinations across the five tasks.

A total of nine hallucinations were observed across the five evaluation tasks. Notably, the GraphRAG-augmented LLaVA was the only model that exhibited zero hallucinations, suggesting that the addition of the retrieved information significantly improved factual stability and contextual alignment. In contrast, LLaVA 1.5 demonstrated the highest number and diversity of hallucinations, including one factual (Task 3), two inferential (Tasks 2 and 4), and one identification error (Task 5), suggesting a tendency to overgeneralize or speculate. Gemini consistently produced identification errors across four tasks (Tasks 1, 2, 4, and 5), typically mislabeling image content or confusing object descriptions. Finally, GPT-4o exhibited a single factual hallucination in Task 5, indicating a generally strong performance, though not immune to inaccuracies. Overall, these findings highlight the importance of structured retrieval mechanisms like GraphRAG for reducing hallucinations in domain-specific, image-grounded tasks. These results are shown in Figure 10.

### 4.9. Runtime Comparisons

**Figure 11:**
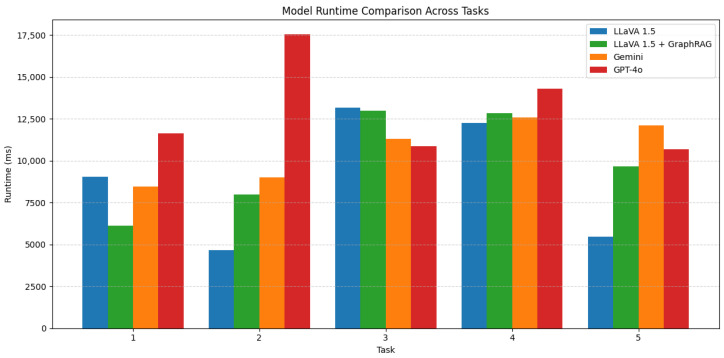
Total runtime comparison across the five tasks.

Comparing total runtime highlights a clear performance gradient across models, with GPT-4o generally incurring the highest computational cost, particularly in Task 2, where its response time peaked at over 17,000 ms. This is consistent with its large-scale architecture and the additional processing required for high-fidelity output. In contrast, the base LLaVA 1.5 model often produced the fastest responses, averaging lower runtimes across most tasks, albeit at the cost of increased hallucination frequency. Gemini showed moderate runtime efficiency, but its performance remained variable, likely due to unstable response generation in visually complex prompts. Meanwhile, LLaVA + GraphRAG, while often slower than the base LLaVA model due to the overhead of graph-based retrieval, remained within acceptable boundaries, especially given its strong gains in factual accuracy and hallucination avoidance. These results suggest a clear trade-off between inference speed and response quality, with the GraphRAG-augmented model offering a favorable balance for domain-specific applications where reliability is prioritized over raw speed. These results are shown below in Figure 11.

### 4.10. Discussion

The above results provide some interesting insights into the positive impact the addition of retrieval technology, specifically GraphRAG, can have on an LLM’s response generation capabilities. Qualitatively comparing the augmented model’s responses to those of the core LLaVA model highlight that in many of the tasks, the addition of the expert knowledge helped to improve the legitimacy, accuracy, and therefore validity of the answers. Although the core model could perform adequately on most tasks, it did generate some questionable information, and in one instance, it hallucinated a response. When evaluating and comparing to the commercial models Gemini and GPT-4o, which were hypothesized to flourish on such tasks, there were some observed problems. Gemini consistently misidentified the object within the image, leading to the generation of much less relevant information within its responses to the input prompts. GPT-4o performed well overall, aside from one small hallucination within the final response, showcasing the depth of its extensive training data. The key finding of this research, suggested from the results, is that the addition of GraphRAG technology into the core LLaVA model can successfully augment responses by providing the LLM with this additional external information relevant to the query. As there exists previous literature describing the successful addition of GraphRAG to other language models and other domains [58,59], this study served to trial how it would meld with and impact the qualitative performance of the LLaVA multimodal LLM in the context of orbital debris-related queries. Significant research efforts in the field have been dedicated to testing and applying AI technologies to facilitate the detection of RSOs. This research suggests that continuing this by utilizing language models may be a logical next step, as most are able to relatively easily decipher an object from an image with training data alone. With the addition of RAG elements to provide expert context regarding detection, such as salient features for extraction, utilizing LLMs could provide researchers in the field with a new approach to solving this problem.

The application of LLMs is rapidly growing in many fields of research, and this study lays a foundation for their application to all areas of orbital object detection. In a high-stakes domain like this, correct information can be considered critical, and if the addition of retrieval technologies can solidify trust, even slightly, this provides reason to consider them when building language models to augment tasks in the domain. The results of the current study confirm that GraphRAG is an appropriate way to provide this additional information to a language model, as observed in the previous literature surrounding it. Emerging research in the field of RAG also has potential for application to debris detection. Zhan et al. [60] proposed Multimodal RAG (MM-RAG), a method that jointly embeds both text and image information, allowing for the retrieval of both at the point of inference to inform multimodal reasoning. This allows for relevant visual domain information to augment the model’s response, as opposed to solely textual information. In the context of debris detection, retrieving images of debris that contained successful detection boundings or other relevant information may help the model to assist in any queries surrounding both the detection of debris and the generation of any related explanations or descriptions. Furthermore, incorporating this into a graph-based retrieval engine is described by Lee et al. [61], who introduced Multimodal Reasoning with Multimodal Knowledge-Graph (MR-MKG). The authors proposed a method that leverages multimodal knowledge graphs (MMKGs) to embed relevant images alongside textual expert information, resulting in a graph that contains nodes representing both documents and images, in contrast to a typical GraphRAG system where nodes represent text-only documents. Adapting the framework proposed in the current study to include salient methods from emerging research may have significant benefits for further developing knowledge retrieval technologies for orbital debris-related activities.

Additionally, the integration of the GraphRAG had little impact on model runtime; for most queries, this was similar for both models. In some instances, the augmented model took longer to run due to the increased size of the input prompt when the retrieved information was appended; however, this was at most increased by a few seconds. Nonetheless, the corpus for the current study was relatively small, and as it increases, so too will computational complexity and load. As such, this is a crucial consideration for any further work incorporating a GraphRAG system for debris-centric research. Finally, the performance of Gemini and GPT-4o used for comparison provides an interesting direction for future research to compare which of these models is best-suited to serve as the underlying LLM for the GraphRAG system for orbital debris. Although such models are not open-source or as easily accessible as LLaVA, there are methods of utilizing them through APIs, which would allow for the integration of the GraphRAG system from the current research. As these cutting-edge models are generally more developed, they may be a better core component for the framework than LLaVA, although this is something that must be analyzed and verified.

## 5. Directions for Future Research

Overall, this research forms the foundation for developing LLM-centric technologies for debris detection. However, there are many challenges left to overcome in order to implement this thoroughly. Firstly, although benchmarks aimed at assessing multimodal RAG performance have been proposed [62,63], as this has not been attempted in the domain of debris detection before, there is a lack of quantitative benchmarks available to assess the performance of the model. This resulted in the current study assessing performance based on a qualitative review of the model’s generated output, which is inherently subjective and may be open to bias [64]. Establishing a test dataset, especially a thorough benchmark for assessing multimodal language model performance, would require the curation of appropriate real-world images of debris in situ, which are not readily available. The research team has, however, been in talks with a stakeholder in the domain with access to such data, and the curation of this dataset to build a testing benchmark for the field is one of the immediate future aims of this work. Establishing this benchmark would make comparisons between various base LLMs viable, allowing for analysis of which may be best suited to the system.

Furthermore, the current preliminary study used a small corpus of papers, as the aim was solely to confirm the theoretical utility of the system. A much larger corpus will be necessary to provide the model with significant amounts of information, especially in such a complex domain. Adding additional papers will increase both the computational complexity and required resources, leading to increased inference times. Compared to the core LLaVA model, the results indicate that in many of the tasks, the addition of the RAG system did not affect the computation and inference runtimes significantly, despite the increased complexity. This is possibly a result of the retrieved context reducing ambiguity in the prompt, as discussed by Chan et al. [65], which would allow the model to generate a more concise response and decrease computation time due to the reduction in decoding steps necessary during execution. However, it is also possible that this is merely the result of some variance in run-to-run speed, and more thorough testing of this is necessary in any following work. If the number of papers was increased significantly, there would be an increased search space for similarity matching. When querying the graph, the model computes the similarity between the query vector and the embedding of each node in the graph; with three nodes, this involves three cosine similarity calculations. If the corpus was increased to 100 papers, that would result in 100 calculations, indicating a linear increase in computation. Furthermore, this would also impact the complexity of the ranking process, which increases logarithmically, as well as both graph traversal speed and prompt assembly time. Balancing performance and efficiency is therefore a key consideration in all future work [25].

Finally, further consideration of how this paper’s summaries and relationships are generated may be beneficial. Currently, these are produced by GPT language models, which, while effective at producing the general overviews of papers and how they link to one another, may omit some critical information relevant to more complex tasks requiring semantic retrieval. This may also overlook more nuanced connections between the papers, indicating that considering some alternative approaches could be valuable. Future iterations could incorporate structured summarization techniques, such as guided abstraction methods [66], or attempt to include relation extraction models to improve the interconnection quality [67]. Ultimately, by improving the quality of the generated summaries and relationships, the retrieval abilities of the model are also developed, in turn improving the accuracy and reliability of the output.

## 6. Conclusions

This study has set out to improve orbital debris identification by adapting the LLaVA multimodal language model through integration with a GraphRAG system backed by a Neo4j-enabled knowledge graph of academic literature. The approach has enhanced the model’s ability to reason over image and text inputs while grounding its responses in verifiable, domain-specific sources. In doing so, it has addressed several common limitations of retrieval-augmented generation systems, including hallucination and contextual irrelevance. For example, the augmented model is able to retrieve and incorporate findings from a specific paper relevant to an input prompt, whereas the base LLaVA model generated a speculative response. Further comparisons to leading commercial models also resulted in similar findings with regard to specific tasks and prompts. This suggests that the proposed technique meaningfully improves both the interpretability and reliability of model outputs in the context of various orbital debris-related tasks. While thorough testing to confirm this improvement in a quantitative sense is not immediately possible, this informs a critical next step for follow-up research to curate and utilize a domain-specific multimodal dataset and benchmark tests. Other directions for future research include refining the summarization and relationship extraction pipelines, building a more comprehensive corpus of external information relating to debris detection, and scaling this in a way that is also cognisant of computational complexity and power. Nonetheless, this preliminary study can be considered successful based on the above directions, clearly paving the way for follow-up research into the problem. The findings of this research posit that domain-specific structured knowledge integration successfully assists in LLM generation for multimodal tasks in the orbital debris domain.

## Figures and Tables

**Table 1:** Selected test queries.

Query	Task(s)
Is this an image of orbital debris?	Image confirmation
What is this object?	Image description
Is this an image of orbital debris? If so, what is an example of a relevant academic paper relating to the detection of such debris? For this paper, discuss the content.	Image confirmation/knowledge retrieval
For the object in this image, describe some of the risks associated with such objects.	Knowledge retrieval/advanced reasoning ^†^
What is the current state of the research surrounding these objects?	Knowledge retrieval/advanced reasoning ^†^

^†^ Examples of multi-hop reasoning style tasks.

**Table 2:** Responses to the query “Is this an image of orbital debris?”.

Model	Response	Runtime (ms)
LLaVA 1.5	Yes, the image is of an object in space, possibly an orbital debris or a satellite, floating in the blackness of space. The object is small and appears to be the only one in the scene, making it stand out against the vastness of the universe.	9051.97
LLaVA 1.5 + GraphRAG	Yes, the image is of orbital debris.	6114.53
Gemini	Based on the visual characteristics, this image likely shows the Rosetta spacecraft, and not orbital debris. Here’s why: Defined Structure: The object in the image has a clear, structured form with recognizable components like solar panels and a central body. Orbital debris is typically shown as irregular fragments and pieces. Rosetta Spacecraft: The shape and configuration in the image are consistent with images of the Rosetta spacecraft, which was a European Space Agency mission to study Comet 67P/Churyumov–Gerasimenko.	8450.10 ^1^
GPT-4o	This image does not appear to depict orbital debris. Instead, it shows a well-structured, intact satellite. The object is symmetric, equipped with solar panels, antennae, and other instrumentation typical of operational satellites. Orbital debris usually consists of broken fragments, irregular shapes, or defunct equipment, not complete functional satellites.	11,630.41 ^1^

^1^ User-timed, as model runtimes are not visible/accessible.

**Table 3:** Responses to the query “What is this object?”.

Model	Response	Runtime (ms)
LLaVA 1.5	The image features a small, square-shaped object floating in the middle of a black background. The object appears to be a satellite, which is a man-made device designed to orbit the Earth and perform various functions such as communication, navigation, and scientific research. The satellite’s presence in the image suggests that it is either in the process of being launched or has recently been deployed into space.	4645.62
LLaVA 1.5 + GraphRAG	The object in the image is a small satellite or space debris. It is located in the middle of a black background, which makes it stand out. The image is a close-up of the satellite, allowing for a detailed view of its structure. The satellite appears to be relatively small compared to the vastness of space, but its presence in the image highlights the importance of monitoring and tracking such objects to ensure the safety of space operations.	7992.51
Gemini	The object in the image is likely the Rosetta spacecraft. Rosetta was a European Space Agency (ESA) mission launched in 2004 to study Comet 67P/Churyumov–Gerasimenko. It was the first spacecraft to orbit a comet and the first to deploy a lander (Philae) onto a comet’s surface. The spacecraft had distinctive features: Large Solar Panels: Rosetta had two very large solar panel wings, necessary to generate power at the comet’s distance from the Sun. These are visible in the image. High-Gain Antenna: A large dish antenna was used for communication with Earth. This is also a prominent feature in the image. Box-like Structure: The main body of the spacecraft had a central, box-shaped structure where instruments and subsystems were housed. The mission concluded in September 2016 with a controlled impact of the Rosetta orbiter onto the surface of the comet.	9021.04 ^1^
GPT-4o	The object in the image appears to be a satellite in space. It has identifiable features such as solar panels, antennas, and structural components typical of Earth observation or communication satellites. Based on the appearance, it may be a small or medium-sized satellite used for commercial, scientific, or military purposes. If you have more context (e.g., mission name, where the image is from), I can help identify it more specifically—especially if it’s related to a known satellite constellation or project like CubeSats, SPARK, or others.	17,530.64 ^1^

^1^ User-timed, as model runtimes are not visible/accessible.

**Table 4:** The responses to the query “Is this an image of orbital debris? If so, what is an example of a relevant academic paper relating to the detection of such debris? For this paper, discuss the content”.

Model	Response	Runtime (ms)
LLaVA 1.5	Yes, the image is an image of orbital debris, which is a space debris or a fragment of a spacecraft that remains in orbit around the Earth. An example of a relevant academic paper relating to the detection of such debris is “Orbital Debris Removal: A Review of the Current State of Technology and Potential Future Approaches” by J. A. S. Lee, published in the Journal of Spacecraft and Rockets in 2013. This paper discusses the current state of technology and potential future approaches to remove orbital debris from Earth’s orbit. It reviews various methods, including active and passive removal, and discusses the challenges and limitations of each approach. The paper also highlights the importance of addressing the issue of orbital debris, as it can pose risks to both spacecraft and the environment. The paper concludes with a call for further research and development of effective orbital debris removal technologies.	13,154.57
LLaVA 1.5 + GraphRAG	Yes, the image is of orbital debris. An example of a relevant academic paper relating to the detection of such debris is “Detecting, Tracking and Imaging Space Debris” by D. Mehrholz L. Leushacke W. Flury R. Jehn H. Klinkrad M. Landgraf. This paper focuses on the current state of space debris and the potential hazards it poses. The authors discuss the efforts made by the FGAN Research Institute and the European Space Operations Centre (ESOC) in tracking and studying this debris. The paper begins by noting that the man-made space debris environment has been created by space activities since the launch of the first artificial satellite in 1957. The authors also highlight the importance of space debris monitoring and the challenges faced in detecting, tracking, and imaging space debris. The paper presents a comprehensive overview of the problem and the methods used by certain institutions for detection, tracking, and imaging of space debris.	12,977.28
Gemini	Based on the visual characteristics, the image most likely shows the Rosetta spacecraft, identifiable by its large solar panels and central structure. It is not typical orbital debris. A relevant academic paper on debris detection is “Serendipitous Detection of Orbital Debris by the International Liquid Mirror Telescope: First Results” by наблюдение, et al. (2024). This paper details the ILMT’s initial observations, detecting streaks from orbiting objects. Notably, 48% of the detected streaks did not match known cataloged objects, indicating a significant population of uncatalogued debris. The ILMT demonstrated the ability to detect objects down to magnitude 14.5, with potential for fainter detections. The study highlights the incompleteness of current debris catalogs and the value of zenith-pointing telescopes for monitoring the orbital environment.	11,285.22 ^1^
GPT-4o	The image shows an intact satellite, not orbital debris, which typically appears fragmented or irregular. A foundational paper on debris detection is Liou and Johnson’s “Risks in Space from Orbiting Debris” (Science, 2006). It quantifies debris populations and highlights how small fragments travelling at orbital speeds pose serious threats. The paper introduces the Kessler Syndrome, where collisions create more debris, increasing the risk of further collisions. It also outlines mitigation strategies such as post-mission disposal and de-orbiting techniques. Detection methods include ground-based radar and optical systems, effective for cataloguing debris larger than 10 cm in low Earth orbit and 1 m in geostationary orbit. However, tracking smaller debris remains a challenge. The paper underscores the importance of international coordination to minimise debris generation. While primarily focused on physics and policy, it laid groundwork for later developments in AI-assisted detection and real-time tracking technologies, which now play a growing role in orbital debris monitoring.	10,872.72 ^1^

^1^ User-timed, as model runtimes are not visible/accessible.

**Table 5:** Responses to the query “For the object in this image, describe some of the risks associated with such objects”.

Model	Response	Runtime (ms)
LLaVA 1.5	The object in the image is a small satellite or spacecraft, which is floating in the dark sky. There are several risks associated with such objects, including the possibility of collision with other objects in space, such as satellites or debris. Collisions can cause damage to the satellite or spacecraft, potentially leading to malfunction or even destruction. Additionally, the presence of debris in space can pose a risk to the satellite or spacecraft, as it may collide with debris and cause damage. Furthermore, the satellite or spacecraft may also be at risk of being hit by meteoroids or other celestial objects, which can cause damage or even destroy the satellite or spacecraft. To mitigate these risks, it is crucial to have proper monitoring and tracking systems in place to ensure the safety of spacecraft and satellites in orbit.	12,235.70
LLaVA 1.5 + GraphRAG	The image features a small object, possibly a satellite or space debris, floating in the blackness of space. These objects can pose risks to both human activities in space and the overall safety of space operations. Some of the risks associated with such objects include: 1. Collisions: Collisions between space debris and operational satellites or other spacecraft can cause damage or even complete destruction of the affected objects. This can lead to costly repairs or loss of valuable data, as well as potential disruptions to communication, navigation, or scientific missions. 2. Debris Field Growth: The accumulation of small debris can create a chain reaction, leading to the creation of larger debris objects through collisions. This can further increase the risk of future collisions and exacerbate the problem. 3. Hazard to Human Spaceflight: The presence of debris in the vicinity of spacecraft or space stations can pose a risk to human spaceflight, as it increases the likelihood of damage to the spacecraft or the space station itself.	12,848.58
Gemini	Based on the image and the understanding that it likely depicts the Rosetta spacecraft, here are some of the risks associated with such objects in space: Operational Risks: Power Management: Rosetta relied on solar panels for power. As it moved further from the Sun on its journey with Comet 67P/Churyumov-Gerasimenko, the amount of solar power available decreased significantly. This limited the operation of its instruments and eventually led to the end of the mission. Distance and Communication: The vast distances involved in space missions create challenges for communication. As Rosetta moved further away, the signal strength weakened, and there could be periods of blackout, such as during solar conjunctions when the Sun is between the Earth and the spacecraft. Harsh Space Environment: Spacecraft are exposed to extreme temperatures, radiation, and micrometeoroid impacts, which can degrade their systems over time. Rosetta endured over 12 years in this harsh environment. Orbital Mechanics and Navigation: Navigating near a comet with a weak and irregular gravitational field, as was the case with 67P, is complex. Rosetta required frequent trajectory adjustments to stay on course, accounting for the comet’s gravity, solar radiation pressure, and even aerodynamic drag from the comet’s coma. Landing Challenges: If the object in the image is related to a landing attempt (like the Philae lander on Rosetta), there are significant risks associated with landing on an unknown and potentially hazardous surface. This includes the lander tipping over, not anchoring properly (as happened with Philae), landing in an unfavorable location (e.g., in shadow, affecting power), or being damaged by surface features. Spacecraft Longevity and Aging: Over long durations, spacecraft components degrade, increasing the risk of failure. Rosetta’s instruments and systems experienced wear and tear throughout its mission.	12,580.45 ^1^
GPT-4o	Satellites like the one shown in the image present several risks that are important to consider in the context of orbital safety and sustainability. One of the most significant concerns is the potential for collisions in low Earth orbit, where traffic is dense and even small fragments can cause catastrophic damage due to the high velocities involved. A single collision can generate thousands of debris fragments, which in turn increase the likelihood of further collisions—a phenomenon known as the Kessler Syndrome. This poses a serious threat to both current and future missions. Another major issue is related to the end-of-life phase of satellite operations. If a satellite is not properly deorbited or moved to a designated graveyard orbit after completing its mission, it remains in orbit as a defunct object. This contributes to long-term congestion and increases the risk of collision with operational spacecraft. Additionally, there is the danger of losing control over a satellite, whether due to power system failures, damage to solar panels, attitude control issues, software errors, or cyberattacks. A non-responsive satellite becomes an unpredictable hazard, potentially drifting across multiple orbital paths. There is also the geopolitical dimension of risk, particularly the potential for satellites to become targets in conflicts involving anti-satellite (ASAT) weapons. The deliberate destruction of a satellite in orbit creates a large volume of debris, further exacerbating the already critical space debris problem. Lastly, satellites that malfunction or are improperly configured may cause radio frequency interference, which can disrupt communication links, satellite navigation signals, or Earth observation data collection, affecting both civilian and military systems. These risks underline the importance of rigorous space traffic management, responsible satellite design, and international cooperation to ensure the long-term sustainability of near-Earth space.	14,304.82 ^1^

^1^ User-timed, as model runtimes are not visible/accessible.

**Table 6:** The responses to the query “What is the current state of the research surrounding these objects?”.

Model	Response	Runtime (ms)
LLaVA 1.5	The current state of research surrounding these objects, which are small black objects floating in space, is still ongoing. Scientists and researchers are actively studying these objects to understand their properties, origins, and potential implications for our understanding of the universe. The study of these objects can provide valuable insights into the formation and evolution of celestial bodies, as well as the processes that shape and influence the cosmos. As a result, ongoing research in this area is crucial for advancing our knowledge of the universe and its various components.	5455.89
LLaVA 1.5 + GraphRAG	The current state of research surrounding space debris involves various institutions and organizations working together to detect, track, and study the problem. The current research papers provide different approaches to addressing the issue of space debris, such as using deep learning algorithms, radar observations, and traditional radar tracking. These papers contribute to the broader field of automated space object detection and tracking, and their methodologies could complement each other in advancing the field.	9677.46
Gemini	The Rosetta mission concluded on 30 September 2016, with a controlled landing on Comet 67P/Churyumov-Gerasimenko. 1 Currently, the Rosetta spacecraft is no longer operational as it impacted the comet’s surface at the end of its mission. However, the vast amount of data collected by the orbiter and the Philae lander continues to be analyzed by scientists worldwide. Research is ongoing, focusing on the wealth of information gathered about the comet’s composition, structure, and behavior as it orbited the Sun. This includes studying the organic molecules discovered, the unique characteristics of its water compared to Earth’s, and the insights into the comet’s formation and its potential role in the early solar system. The data is crucial for understanding comets in general and their contribution to the evolution of our solar system. 2 While there are no active missions directly studying Comet 67P at this moment, the legacy of Rosetta persists through the continuous scientific investigation of its findings.	12,089.08 ^1^
GPT-4o	The object in the image appears to be a SPARK (Space-based Platform for Advanced Reconnaissance and Knowledge) satellite. Research on such small satellites focuses on their use for space-based surveillance, Earth observation, signal intelligence, and rapid deployment constellations. Current work includes improving their onboard autonomy, miniaturised sensors, and communication systems, as well as addressing challenges like space debris avoidance, secure data handling, and cooperative satellite swarms. There’s increasing interest in using AI for onboard processing to reduce reliance on ground stations.	10,672.60 ^1^

^1^ User-timed, as model runtimes are not visible/accessible.

## Data Availability

The SPARK dataset utilized in the study is available at https://cvi2.uni.lu/spark-2022-dataset/, courtesy of Musallam et al. [45] (accessed 16 April 2025). The code developed during this project will be made available through a specific GitHub repository. Specific requests can be made to the research team by contacting them via e-mail.
